# The Combined Spectral Response of a MEMS Metamaterial Absorber for the Mid-IR and Its Sub-Wavelength Fabrication Residual Array of Holes

**DOI:** 10.3390/ma16124278

**Published:** 2023-06-09

**Authors:** Reinoud F. Wolffenbuttel, M. Amir Ghaderi

**Affiliations:** 1 Laboratory for Electronic Instrumentation, Department of Microelectronics, Delft University of Technology, 2628 CD Delft, The Netherlands; 2 Infineon Technologies, Am Campeon 1-15, 85579 Neubiberg, Germany; ghaderi.m.amir@gmail.com

**Keywords:** metamaterial absorber, surface micromachining, CMOS compatibility, under-etched metasurface

## Abstract

Metasurface coatings on a free-standing SiN thin film membrane are fabricated on a Si substrate using masked lithography and CMOS-compatible surface micromachining. The result is a band-limited absorber for the mid-IR, which is part of a microstructure that is attached to the substrate by long and slender suspension beams to provide thermal isolation. As a residual of the fabrication, the regular pattern of sub-wavelength unit cells of 2.6 μm side length, which defines the metasurface, is interrupted by an equally regular array of sub-wavelength holes of 1–2 μm diameter and at 7.8–15.6 μm of pitch. This array of holes is essential for enabling access of the etchant and attack of the underlying layer during fabrication, which ultimately results in the sacrificial release of the membrane from the underlying substrate. As the plasmonic responses of the two patterns interfere, a maximum is imposed on the hole diameter and a minimum on the hole-to-hole pitch. However, the hole diameter should be sufficiently large to allow access of the etchant, while the maximum spacing between holes is set by the limited selectivity of the different materials to the etchant during sacrificial release. The effect of the parasitic hole pattern on the spectral absorption of a metasurface design is analyzed by simulations of the responses of combined holes–metasurface structures. Arrays of 300 × 180 μm^2^ Al-Al_2_O_3_-Al MIM structures are mask-fabricated on suspended SiN beams. The results show that the effect of the array of holes can be disregarded for a hole-to-hole pitch larger than 6 times the side length of the metamaterial until cell, while the diameter of the hole should remain smaller than about 1.5 μm, and their alignment is critical.

## 1. Introduction

Silicon has remained the prevalent mainstream material in micro-electro mechanical system (MEMS) technology-based photonics, despite some of the compelling benefits of other materials, such as low-cost transparent substrates based on plastics and combined with patterned polymer layers [1]. The advantage of elastic structures for use in applications such as foldable implantable microsystems would be lost when merged with the rigid silicon substrate. Therefore, sensors and systems on plastic have developed separately from mainstream MEMS. Other optical platforms have emerged because of one of the material limitations of silicon. Unlike silicon, lithium niobate offers the advantage of piezo-electricity and has become an important optical platform. These platforms can still benefit from MEMS technology by deposition of a lithium niobate layer on top of a silicon wafer [2]. Similar technologies are available for the deposition of a top layer of an optical material with a sufficiently small bandgap to have efficient photon detection in the infrared (IR) spectral range [3], or materials on a silicon wafer with a direct bandgap for efficient light emission [4,5]. These are complex, but viable, techniques for adding the missing capabilities to a silicon microsystem. Despite these limitations, silicon is generally considered a very acceptable material for use as an optical platform in the visible spectral range. Rather than its optical properties, the decisive advantage of silicon is in its compatibility with CMOS processing, which is the main reason for adding thin films of materials with specific transduction properties to the silicon wafer [6].

Efficient direct electron–hole pair generation by photon absorption is possible in silicon in the 300–1100 nm spectral range because of its indirect bandgap at 1.12 eV. However, the detection of radiation in the mid-IR spectral range is a particularly desirable property from an application perspective. Photons in this part of the spectrum do not supply sufficient energy for electron–hole pair generation, while the deposition of a crystalline layer of a material with low bandgap on top of a silicon wafer, as in [3], is challenging to achieve in a CMOS-compatible fashion. Therefore, a microsystem based on a silicon thermal detector that is coated with an appropriate absorber is generally considered more practical [7]. Such an IR detector is referred to as a microbolometer and is composed of a temperature sensor with a top coating for efficient optical absorption of incident radiation in the intended spectral range [8,9].

The absorbed IR radiation results in accumulated heat, which is partly stored in the microstructure, while the remainder is released. This heat balancing results in an increase in temperature relative to ambient temperature, which is measured using a thermo-electric effect. Ambient heat loss should be minimized for achieving high sensitivity, which leads to a suspended structure for minimum heat loss. The suspension beams should be of a material of low thermal conductivity. In extremely demanding applications, the structure is contained within a vacuum enclosure to minimize heat loss by convection, and cooling is applied for reducing detector noise [8]. The temperature dependence of an electrical resistor that is integrated in the absorbing membrane is the most common approach for measuring the temperature increase, which however could be a source of parasitic heat injection by dissipation (self-heating). In our work the Seebeck voltage in a thermocouple that is integrated on the suspension beams is used as a direct measure of the temperature difference between absorber and substrate [10].

The absorber should be designed for a high absorption coefficient, α, in the spectral range imposed by the application and should preferably be non-absorbing outside that spectral range for providing maximum sensitivity and spectral selectivity. However, the usual silicon-compatible materials, such as silicon-oxide (SiO_2_), polysilicon (poly-Si), or silicon-nitride (note that the material composition is usually non-stoichiometric, and SiN or SiN_x_ is used rather than Si_3_N_4_), do not offer opportunities for a design with high absorption in the mid-IR [11]. Approaches based on thin-film interference filters using titanium (Ti) or silicon-carbide (SiC) layers [10,11,12] or porous metal layers [13] are reported. A more recent approach is based on absorption in areas coated with vertically grown carbon nanotubes (CNT) [14,15]. These approaches generally result in wideband absorption, which may be desirable in a general purpose pyrodetector, but often introduce an undesirable out-of-band sensitivity in a sensor system that is intended for spectrally selective operation.

A more flexible approach for spectrally selective absorber design is based on coating with a metamaterial, which is a large 2D array of sub-wavelength unit cells with a fine structure within the cell that is engineered to provide resonance (thus absorption) for an electromagnetic wave of well-defined wavelength. As the interaction between the impinging radiation and a metamaterial primarily depends on the side length of the unit cell and the shape of a pattern within it, and not on the bulk material properties, the spectral absorption can be controlled by the layout design of the unit cell [16,17,18]. The feature size limitations of state-of-the-art clean room equipment for lithography make MEMS technology particularly suitable for the fabrication of such structures for gas sensing by the gas-specific absorption spectrum in the mid-IR spectral range [19].

Coating of an absorber area of up to several mm^2^ with an array of identical unit cells of μm^2^ dimensions, each containing an inner pattern with sub-μm features, justifies the use of the term ‘large-area device’ [11]. E-beam lithography is generally used in metamaterial fabrication for the definition of the lateral shape on a substrate. This technology ensures a faithful reproduction of the features in each unit cell. However, operation is essentially sequential and results in a low throughput. This constraint favors the use of mask-based lithography for such large-area absorbers, despite its higher minimum feature size. UV i-line lithography is commonplace in MEMS fabrication.

Most of the applied work in the literature on metamaterials is driven by a specific performance parameter. Our ambition, however, is to explore and define the design limitations imposed by compatibility with CMOS processing. Our earlier work demonstrates that using masked UV (i-line) lithography instead of e-beam does indeed result in additional shape distortions, but those effects have proven manageable in a design intended for use in the mid-IR [20]. Moreover, we have demonstrated that the preferred use of aluminum for also the metal in the MIM structure results in a spectral absorption curve that is very comparable to that obtained with gold [11]. The core issue considered in this paper is the effect of the surface micromachining used for CMOS-compatible MEMS fabrication on the optical properties of a metasurface.

The intended application is in three-channel selective CO_2_ detection using differential optical absorption spectroscopy (DOAS), with the 4.2–4.4 μm band used for sensing and the 4.0–4.2 μm and 4.4–4.6 μm bands (both containing mainly N_2_O absorption lines) for referencing. The advantages of batch processing in CMOS-compatible fabrication could be decisive for the economic viability of MEMS in potentially high-volume applications, such as air quality measurement. The market size could be considerable in both outdoor (CO_2_ is a well-known greenhouse gas, which calls for large-scale emission monitoring at potential sources) and indoor (avoiding sick building syndrome in poorly ventilated rooms with many people present) applications. This approach has similarities with highly interesting work on a microsystem for CO_2_ detection with both the thermal emitter and detector coated with a metasurface for enhancing the spectral selectivity [21]. In the ultimate device aimed for in this work, spectral selectivity is pursued using DOAS; hence, the metasurface coating is not applied to the thermal emitter.

## 2. Impact of the Thermal Design of a Thermal Detector

**Figure 1:**
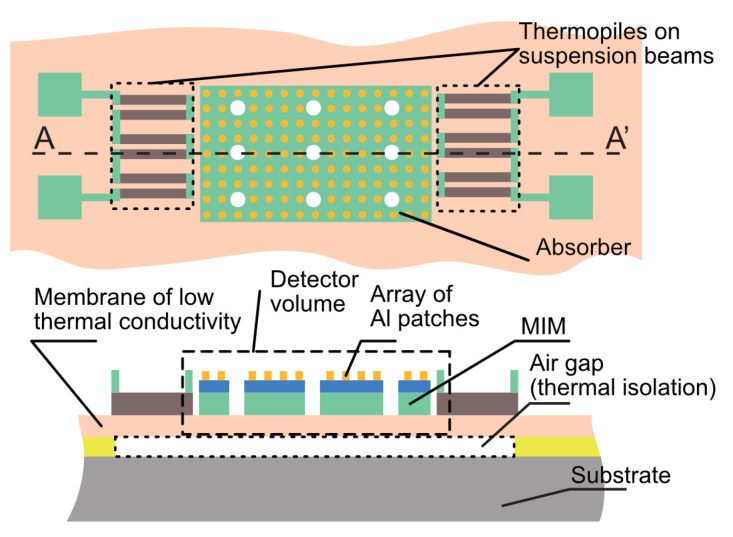
Schematic of a beam-suspended structure in MEMS in top view and cross-sectional view, with a metamaterial coating on the surface of the suspended part for mid-IR absorption and thermopile detectors on top of the suspension beams.

The structure of the MEMS thermal detector fabricated in surface micromachining is shown schematically in Figure 1. In principle there are four parts: (a) the substrate, (b) the detector volume with (c) the absorber, and (d) thermal isolation. Thermal isolation from the substrate is realized using an airgap, while the thin absorber structure is attached to the substrate using slender suspension beams.

The thickness of the airgap is defined during fabrication by the thickness of a deposited sacrificial layer, while the microstructures are patterned in the subsequently deposited structural layer [22]. Polysilicon thermocouples are very suitable for measuring the temperature difference between the suspended area (which is at an elevated temperature due to absorbed radiation) and the substrate (which is acting as heat sink). The top layer of the suspended structure is the absorber, with the spectral absorption defined by the metasurface design. The suspended mass is characterized by its surface area, *S* (m^2^), its thickness, *t* (m), and thermal capacity, *C*_th_ = *c*_p_ × *M* = *c*_p_ × *ρ* × *S* × *t* (J/K), with *M* being the mass (kg), *c*_p_ the specific heat (J·kg^−1^K^−1^), and *ρ* the density (kg·m^−3^). The intermediate layer represents the thermal conductivity, *G*_th_, between the structure and the substrate.

The increase in temperature of the structure relative to the ambient temperature results from the balancing of heat flux entering the structure by absorption and dissipation by devices within the structure (self-heating by circuits), and the heat loss by radiative emission, heat conduction, or convection [7]. In the case of sufficient thermal isolation and no flowing air, the heat generation by absorbed mid-IR radiation is mainly transported to the substrate by thermal conduction through suspension beams. These beams are designed to have low thermal conductivity, *G*_th_, by using long suspension beams and small cross-sectional areas of a material with low specific thermal conductivity. Exposure to radiation of spectral power *P* (W/m^2^) and disregarding self-heating results in a temperature increase, Δ*T* (K), that is described in simplified approximation by a first-order system response with a low-frequency sensitivity described by (Δ*T*/*P*(λ))_DC_ = α*S*/*G*_th_ (Km^2^/W) and a pole position at τ = *C*_th_/*G*_th_ = *c*_p_*ρSt*/*G*_th_ = *c*_p_*ρt*(Δ*T*/*P*)_DC_. Therefore, the sensitivity is proportional to 1/*G*_th_, and the response time is equal to *C*_th_/*G*_th_ [23].

At given *G*_th_ and material properties, the only remaining design parameter for the tuning of the response time is thickness, *t*. Therefore, high sensitivity and fast response can only be achieved simultaneously when using a miniaturized system with a thin layer in which long and slender suspension beams are realized. This requirement is fully compatible with the capabilities of MEMS technology, which enables the utilization of the thermal isolation provided by air layers that result from the fabrication of fully under-etched microstructures in a silicon-compatible dielectric layer, while these are mechanically attached to the substrate by suspension beams that are etched out of that film. Microbolometers fabricated in MEMS technology with the absorber based on a metasurface have been extensively reported [24,25,26].

The requirements imposed on a microbolometer are not unique. A very similar challenge applies to the MEMS-fabricated hot-wire-based thermal emitter. An electric current passing through a resistive wire results in a temperature profile along the length direction of the wire, with a maximum temperature at the center. Efficient mid-IR emission requires a maximum temperature in excess of about 300–400 °C, while the heated surface should act as a blackbody source (high emission coefficient, ε, which is, at thermal equilibrium, equal to the absorption coefficient, α, in the spectral range of interest. A suspended wire is essential for achieving such a temperature profile. Coating with an appropriate metasurface enables selective emission by design of radiation in only the selected part of the spectrum. The response time of the hot-wire to a modulated excitation current is proportional to the thermal capacitance of the wire. A low thermal capacitance is desirable for enabling a relatively high modulation frequency. Combining these two considerations results in a very similar microstructure, as shown schematically in Figure 1, with the exception that the thermopiles on top of the suspension beam are replaced by metal strips for electrically connecting a power source to the heating resistor in the suspended part. Many applications require a narrowband IR light source. Metasurfaces that are engineered to meet specific absorber specifications for use in narrowband hot wire illuminations are already demonstrated in the literature [27,28].

The microbolometer and the hot-wire emitter share the need for low thermal conductivity to the substrate. The micromachining steps for realizing thermal isolation in a MEMS are applied after the realization of the metasurface. Consequently, the fabrication should be arranged in such a way that the metasurface is either intrinsically resilient to the exposure to microfabrication steps (using a suitable choice of materials), or it is protected in such a way that it survives exposure (by additional layers or by design). Analyzing the approaches for minimizing the interference between fabrication strategy and metasurface operation is a major objective of this paper.

## 3. Analysis of Spectral Absorption in a Metamaterial—Hole Array Structure

In this section, first the metamaterial design is introduced and analyzed, followed by a discussion of the plasmonic response that results from the regularly shaped pattern of holes that is required in surface micromachining. Finally, their interaction is discussed using the COMSOL multi-physics software tool version 5.4 for simulations [29].

### 3.1. The Nominal Metamaterial Unit-Cell Design

**Figure 2:**
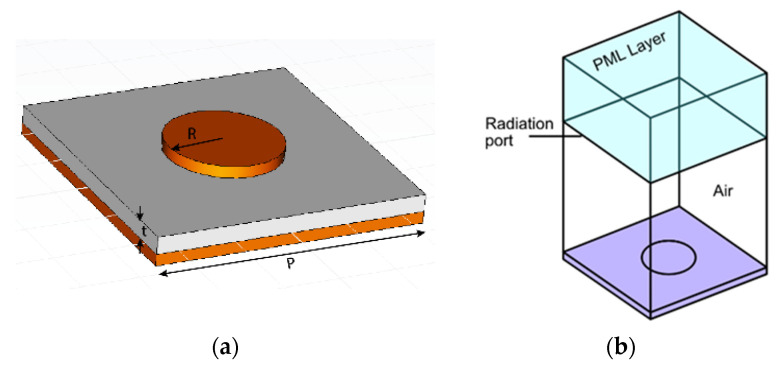
(**a**) Structure of the circular patch in a unit cell and (**b**) the model of this structure as used in COMSOL for absorbance simulations. A perfectly matched layer (PML) was used to back the radiation port, while perfect electric conductor (PEC) and perfect magnetic conductor (PMC) boundary conditions were applied to the sides.

A metamaterial design based on an array of sub-wavelength unit cells, each composed of a metal–insulator–metal (MIM) vertical stack, is shown in Figure 2a. The MIM comprises a metal–based backside reflector, a dielectric layer, and a topside disk-shaped metal reflecting pad (also referred to as a patch) and can be dimensioned as an effective mid-IR absorber [11].

**Figure 3:**
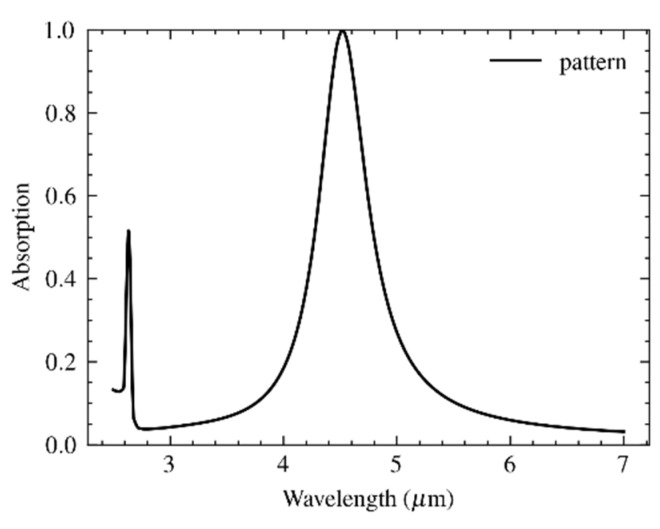
Spectral absorption of the metamaterial design with an Al circular patch of 60 nm thickness and *D* = 2*r* = 1400 nm diameter, on an Al_2_O_3_ spacer of thickness *t* = 150 nm, and a thick Al backside reflector.

A first estimate of the patch diameter can be analytically calculated using a relatively simple equivalent RLC model [30]. This value can be refined by numerical simulations using the model shown in Figure 2b. A perfectly matched layer (PML) was used to back the radiation port. This PML ensures that any reflection from the metamaterial pattern is fully absorbed and avoids calculation errors due to port reflections into the main domain. Moreover, sidewall boundary conditions need to be imposed. The Floquet boundary condition, which is based on periodicity (the *k* vector is continuous over boundaries) is often applied for reasons of accuracy [29]. However, in this paper an alternative approach based on perfect electric conductor (PEC) and perfect magnetic conductor (PMC) boundary conditions was applied to the side planes of the model, which is less calculation intensive and, although it also implies linear polarization, results in acceptable results. The results for a metamaterial design with a unit cell of *P* = 2600 nm side length (pitch) and a MIM structure that is composed of an aluminum (Al) circular patch of 60 nm thickness, a *D* = 2*r* = 1400 nm diameter on a Al_2_O_3_ spacer of thickness *t* = 150 nm, and a thick Al backside reflector are shown in Figure 3.

The simulated spectral absorbance confirmed the design wavelength of peak absorbance at λ_o_ = 4.5 μm and the full width at half-magnitude (FWHM) bandwidth of 500 nm. It should be noted that the analysis so far is about utilizing localized surface plasmon (LSP) resonance. The additional peak at 2.6 μm corresponds to a propagating surface plasmonic (PDP) resonance, which is defined by the periodicity of the unit cell rather than the patch dimensions within the cell [31].

However, the actual MEMS fabrication resulted in a more complex structure, and the fabrication-imposed issues presented needed to be considered in the design stage to prevent a metamaterial with a reduced optical performance in terms of the mid-IR optical absorption. The details of the fabrication flow, the choice of the dimensions of the pattern of holes, and the resulting plasmonic response are described in the next sub-section.

### 3.2. Surface Micromachining and the Plasmonic Response of the Resulting Pattern of Holes

The airgap underneath the structure is an essential part of the thermal isolation of the absorber. Several approaches are possible for achieving air isolation. Bulk micromachining enables the removal of the substrate underneath the absorber by etching, starting from the backside of the wafer at mask-defined parts and progressing though the entire wafer thickness until the frontside is reached. Etching should stop at the frontside of the wafer, thus at the backside surface of the membranes that contain already-integrated CMOS devices. Obviously, these devices should remain, while the membrane material should be highly resilient to the etchant used for through-wafer etching or should be protected during etching.

MEMS-based metamaterials have been successfully fabricated using bulk micromachining from the wafer backside and used for CO_2_ sensing [21]. This technique has the advantage of a non-exposed metasurface during fabrication, as it is located at the frontside of the layers at the moment the membrane is formed, while the frontside does not require a special pattern for providing access to the etchant. The through-wafer etching is a disadvantage, and this technique is generally considered less CMOS-compatible as compared to its alternative, surface micromachining.

In surface micromachining, exposure to the etchant is at the wafer frontside. The position and thickness of the airgap are defined during fabrication by the lateral pattern and thickness of a deposited sacrificial layer. The microstructures are included in the subsequently deposited structural layer and should endure and survive the sacrificial etch step that is applied for undercutting this structure. It is essential that a combination is found between (a) an etchant that efficiently removes the sacrificial layer, and simultaneously (b) a structural material that is resilient to this etchant, as it is intended to withstand the under-etching. This constraint limits the number of options. Although alternative options are available, SiO_2_ is traditionally used as the material for use in the sacrificial layer with wet etching in HF. This implies that any of the materials that are intended to remain should be resilient to the HF etchant [32].

The poly-Si layers used for the thermocouples have an etch rate typically smaller than 0.1 nm/min, while thermally grown SiO_2_ has an etch rate higher than 50 nm/min. Deposited SiO_2_ generally has a higher etch rate in HF depending on the specifics of the deposition process, and the selectivity (etch rate of the sacrificial material/etch rate of the structural material) typically exceeds 10^3^. SiO_2_ is often used as the dielectric spacer in a MIM patch for use in the mid-IR but would simply be removed during sacrificial release. Therefore, the choice of the SiO_2_/HF system for micromachining implies that the MIM design should involve a different dielectric material, and Al_2_O_3_ was used in this work.

This adaptation does not address the attack of the other layers in the MIM structure, which are the Al patch and backplane. Unless a high-concentration HF is used, the Al-based patches are vulnerable to the conventional HF attack in case the sacrificial release takes place in a liquid. A more advanced setup makes use of vapor HF, which features a significantly improved selectivity, while also the problem of ‘stiction’ is avoided [33]. 

These two minor adaptations to the conventional fabrication flow provide a solution to the compatibility issue described. However, a final design constraint remains, which is ensuring complete release of the microstructure by removal of the entire underlying sacrificial layer. In principle, under-etching is achieved by lateral access of the etchant from the sidewalls and etch propagation through a narrow lateral channel (having a width equal to the thickness of the sacrificial layer). Typically, tens of micrometers of SiO_2_ should be removed sideways, while the attack of the structural layer should be limited to several nanometers. Although the lateral etch rate of SiO_2_ is significantly improved when using vapor HF during the sacrificial release, lateral access from the sides is insufficient in a design with a large cross-sectional aspect ratio (length/height), and additional access holes through the structural layer should be included. The diameter of each access hole should be sufficiently large to allow access of the etchant during the sacrificial release, while the distance between adjacent holes is limited by the selectivity of the different materials to the etchant used for sacrificial release.

As a consequence, the regular pattern of sub-wavelength unit cells, already fabricated in the structural layer for defining the metasurface, is interrupted by an equally regular array of holes of (sub)-micron diameter (thus also of sub-wavelength in the mid-IR) and typically at a 10–20 μm pitch. This array of holes is an inevitable result of the fabrication, but its effect can be limited by seeking a solution in terms of optimum dimensions. The array of sub-wavelength diameter access holes results in a plasmonic response. This effect has already been reported in the literature in a metal-coated reflecting surface with an array of sub-wavelength holes [34] and is sometimes referred to as Fano resonance when operating in transmission [35].

**Figure 4:**
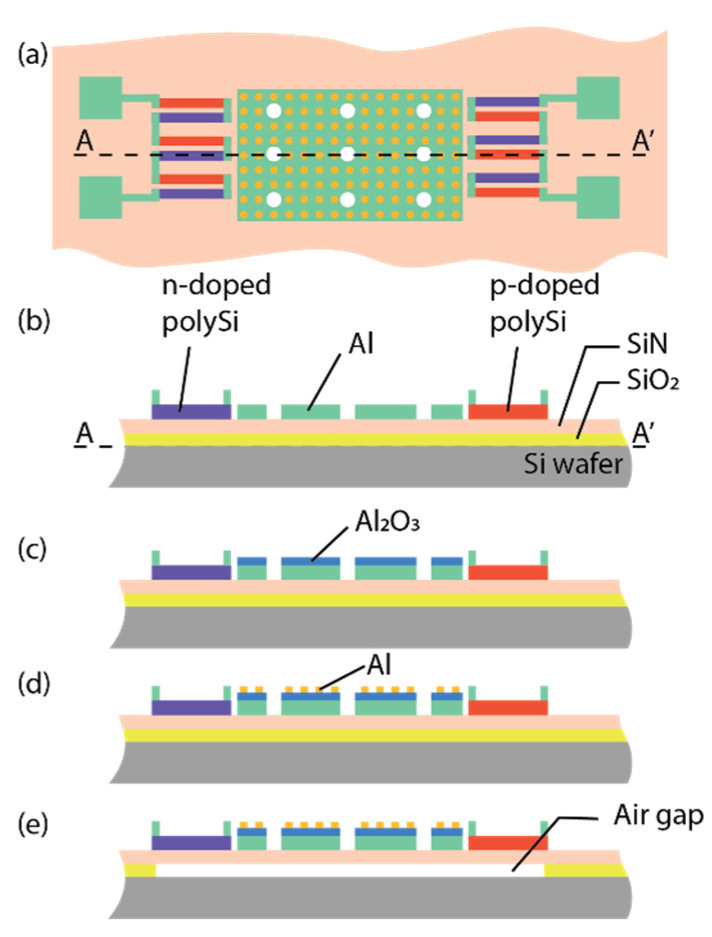
Fabrication process: (**a**) device schematic, and (**b**) deposition of SiO_2_, SiN, and poly-Si forming n- and p-doped regions, etching poly-Si; and deposition of Al and patterning for IC and metamaterial back reflector; then (**c**) sputtering of the Al_2_O_3_ spacer, (**d**) patterning of metamaterial disk patch, Al deposition, and lift-off; and finally (**e**) vapor HF removal of the SiO_2_ sacrificial layer, dicing, and wire-bonding.

The flow of the surface micromachining process and the materials used are shown schematically in Figure 4. Silicon wafers (4 inch) were used as the substrate. Small linear arrays of suspended membranes with metasurface coating were fabricated, each with an area of 300 × 180 μm^2^. The metallic patch design was based on circular Al disks of 1400 nm diameter. As a first step, a thin SiN layer and a SiO_2_ sacrificial layer of 1 μm were deposited, followed by the deposition of a SiN layer of 500 nm thickness for the membrane and the poly-Si layer for the thermopiles. Implantation was applied for realizing n- and p-doped regions in the thermopiles. Subsequently, masked lithography was applied for laterally defining the suspension beams and poly-Si strips for forming the thermocouples. In the next step (b), 670 nm of AlSi was deposited and patterned for defining the electrical interconnections and metamaterial backside reflector. In the next step (c), the dielectric spacer in Al_2_O_3_ of 150 nm thickness was sputtered. Subsequently, the metamaterial pattern was transferred using i-line lithography (Wafer stepper PAS5500, ASML) on negative resist (nLof2020). In the next step (d), 65 nm of Al was sputtered and lifted off in an ultrasonic bath of PGMEA. In the final step (e), the sacrificial oxide layer was etched in vapor HF to release the SiN membrane, followed by dicing and wire bonding. Note that the sacrificial layer was not explicitly patterned to lateral definition of the sacrificial layer, and consequently the membrane contours were slightly under-etched.

The challenge was to define the dimensions of an array of access holes (in terms of hole-to-hole pitch and hole diameter) that (a) would fit within a contiguous array of unit cells of the metasurface design and that (b) would have a minimum impact on the spectral response of that design. Here, circular patches of 1.4 μm diameter were used, each at the center of a unit cell of 2.6 μm side length. Consequently, the shortest spacing between two adjacent patches was 2.6√2 − 1.4 = 2.28 μm. Initial tests on this surface micromachining process indicated that an array of holes of 2 μm diameter and with a pitch of 7.8 μm was optimum from a technology perspective. The hole diameter should preferably be restricted to about 1 μm to satisfy reasonable tolerance requirements with respect to the minimum spacing with a unit cell.

**Figure 5:**
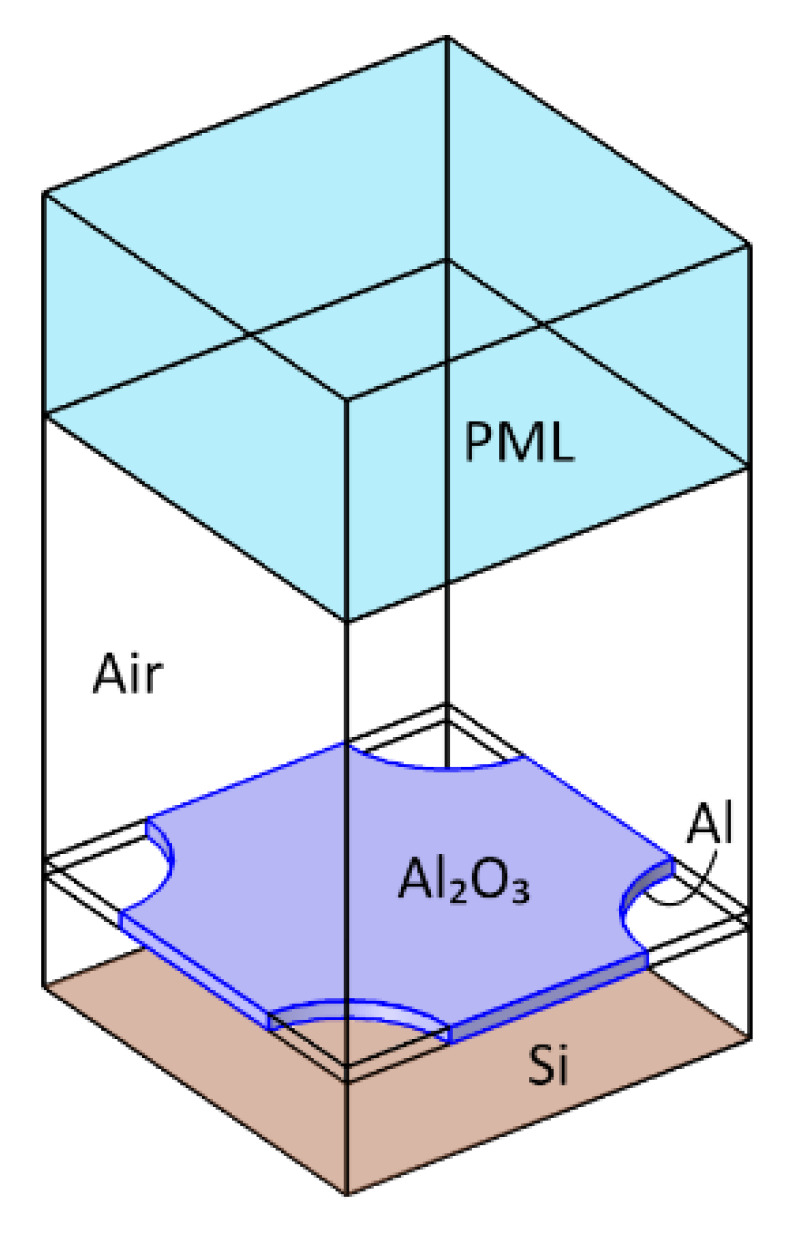
The geometry used for calculations consists of a Si substrate modeled with an impedance boundary condition, air gap, a membrane with a square array of circular holes, and an air domain. A PML layer is placed on the top of the air domain backing the radiation port. The thickness of the PML and air layers are kept at λ_max_/4 and λ_max_/2, respectively, with λ_max_ being the maximum simulated wavelength.

However, the adverse effect of a single non-released structure within the large array on the overall absorber operation was deemed larger than that of a few damaged unit cells, and it was decided to use access holes of 2 μm diameter to ensure that fully under-etched structures would result over the entire membrane area. As a next step, this dimensioning of the array of holes needed to be analyzed from an optical perspective. The model used is shown in Figure 5.

**Figure 6:**
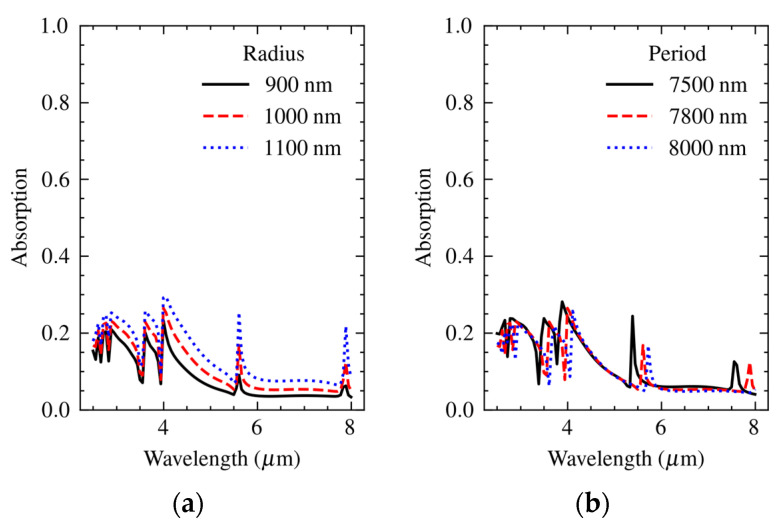
Calculated dependence of the spectral absorption in an array of holes of 7.8 μm pitch on (**a**) hole radius and (**b**) variation of the pitch at nominal hole radius of 1000 nm.

Figure 6a shows the spectral absorption in a rectangular array of holes with a hole-to-hole pitch of 7.8 μm and hole radius in the range from 900 nm to 1100 nm. The resonance wavelengths were at a pitch of 7.8 μm and harmonics of 3.9 μm. Note that the pitch along the diagonal was about 7.8√2 = 11 μm, which explains the peak at about 5.5 μm. The amount of absorption increased with the radius of the holes, while Figure 6b shows that the wavelength of peak absorption shifted with the pitch, which is in agreement with the literature [34]. In the actual structure, the metasurface and the array of holes were merged, resulting in a combined plasmonic response.

### 3.3. Spectral Absorbance of a Metasurface That Is Interrupted by an Array of Holes

**Figure 7:**
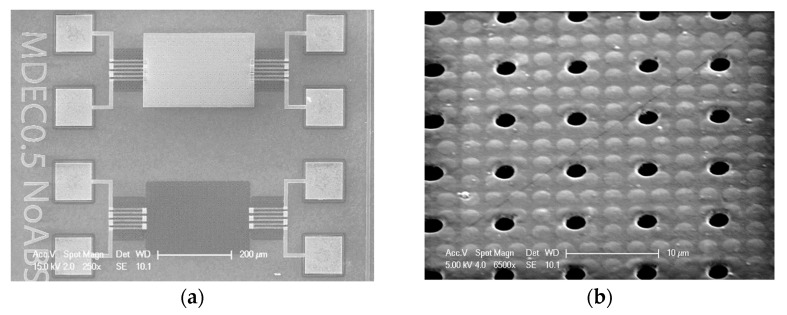
SEM of a chip with metamaterial-based absorber: (**a**) complete bolometer element, and (**b**) magnification. The access holes for the sacrificial etching are visible in the micrographs as black holes.

The pitch of the array of holes is three times the periodicity of the unit cells in the metasurface (7.8 μm/2.6 μm). Both the side length of the unit cell (2.6 μm) and the hole diameter (2 μm) are sub-wavelength when considering the application of absorption in the mid-IR. The hole-to-hole pitch is larger than the design wavelength, λ_o_. A simplified version of the process, shown in Figure 4, was used for fabrication (the steps related to the p-type and n-type doping of the poly-Si layers used for the thermopile were omitted for enabling a focus on the under-etching issue addressed here, but CMOS compatibility was nonetheless strictly observed). The resulting under-etched mass with a metasurface coating over 300 × 180 μm^2^ and suspension beams is shown in the SEM photographs in Figure 7. Figure 7a shows both a coated and an uncoated device (labeled NoABC), which would in principle enable a differential measurement for reducing the effect of temperature gradients within the device.

**Figure 8:**
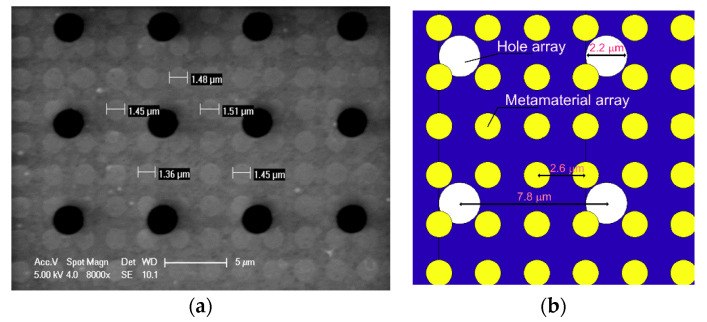
(**a**) SEM with detail of fabricated metamaterials with actual values of the holes with a nominal diameter of 1400 nm and (**b**) drawing of the area covering 2 × 2 access holes.

A detailed image of the metasurface with unit cells and an array of holes is shown in Figure 8a. The unit cells in the SEM images were analyzed using an image processing algorithm in MATLAB. The element-to-element variation likely contributed to a broadening of the absorption band as compared to theoretical expectations.

Figure 8a reveals that the actual access holes were not neatly arranged, and some of the access holes overlapped with the patch in the unit cell area. This was the result of two effects. The first was the over-etching of the access holes (the nominal diameter, as defined by the mask, was 2 μm, but the final diameter was 2.2–2.3 μm). The second was lateral misalignment between the positioning of the mask used for metasurface definition and the mask used for the definition of the array of holes. Consequently, several of the unit cells had an incomplete circular patch, as shown in Figure 8b, which is a drawing of a part of the photograph in Figure 8a.

**Figure 9:**
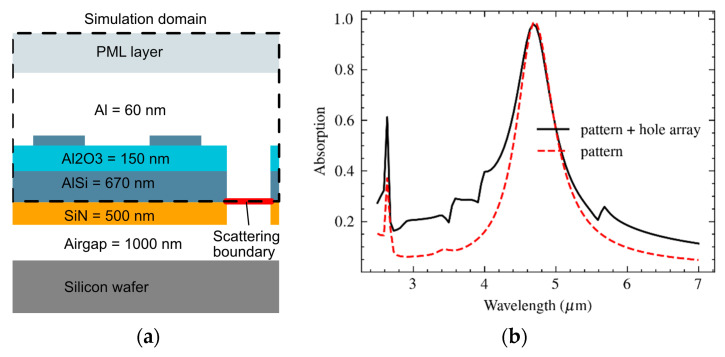
(**a**) Specification of the layers in the model and boundary conditions imposed, and (**b**) the resulting absorption profile of a metamaterial pattern with and without a hole array. The holes in the array have a diameter of 2000 nm and a hole-to-hole pitch of 7800 nm.

The fact that impinging light that is incident onto the access holes is ultimately absorbed in either the silicon substate or the suspended mass is a complication in the modeling already discussed in Section 3.1. This effect reduces the amount of radiation that is available for heating of the suspended volume, while in the experiment, it was assumed that all light was either reflected from or absorbed into the microstructure. This issue was included in the modeling by imposing the scattering boundary condition at the bottom boundaries of the sacrificial etching holes. Consequently, any electromagnetic radiation reaching this boundary was removed. As an unintended but manageable side-effect, the measured temperature increase in the suspended volume was a slightly understated measure for the absorption. The resulting model definition and the calculated spectral absorption are shown in Figure 9.

**Figure 10:**
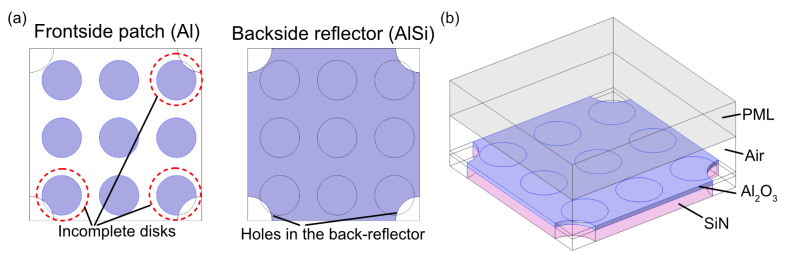
(**a**) Geometry of frontside and backside and (**b**) model of the damaged structure used in simulations.

The results shown in Figure 9b are based on a neatly aligned array of patches of non-distorted shape that is perfectly aligned with an undistorted array of holes. Therefore, it does not include the effect of any shape distortion or overlap in the patch and the hole patterns that are shown in Figure 8. The effect of this damage incurred to the unit cell on the spectral response was analyzed as a next step using the geometry and model shown in Figure 10.

**Figure 11:**
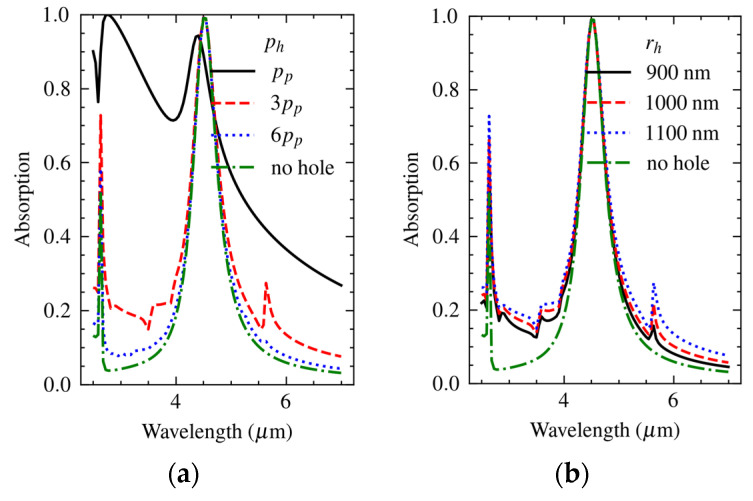
Calculated spectral absorption for the metasurface (**a**) configuration with a superimposed array of holes of *r*_h_ = 1100 nm radius and a pitch *p*_h_ = of 1×, 3×, and 6× the unit-cell side length (=metamaterial pitch, *p*_p_) and (**b**) configuration with a constant hole pitch *p*_h_ = 3*p*_p_ and a hole radius in the range between 900 nm and 1100 nm.

The simulation was carried out for the actual structure (*p*_h_ = 3*p*_p_), but also for *p*_h_ = *p*_p_ and *p*_h_ = 6*p*_p_. The results in Figure 11a show the effect of an increased pitch at a constant hole radius *r*_h_ = 1100 nm, while the effect of hole diameter at a hole pitch *r*_h_ = 3*r*_p_ is shown in Figure 11b.

The results indicated that the effect of an increased hole pitch was more significant compared to that of a reduced hole diameter. Therefore, over-etching is not critical, provided that a sufficient lateral clearance is maintained in the design to avoid damaging the patches. The spectral absorption for *p*_h_ = *p*_p_ was not useful, and an implementation based on a hole-to-hole pitch equal to the side length of the unit cell is not very practical anyway.

The in-band absorption for *p*_h_ = 3*p*_p_ was close to the response of the non-perforated membrane and a further increase in pitch to *p*_h_ = 6*p*_p_ seemed not strictly necessary. However, the out-of-band response was significantly improved by increasing the pitch to *p*_h_ = 6*p*_p_. Therefore, the overall optical performance would be significantly improved when designing for *p*_h_ = 6*p*_p_ and beyond. The fabricated devices were of *p*_h_ = 3*p*_p_, and a significant widening of the bandwidth could be expected, while the short-wavelength threshold of absorption would be at about α = 0.2.

**Figure 12:**
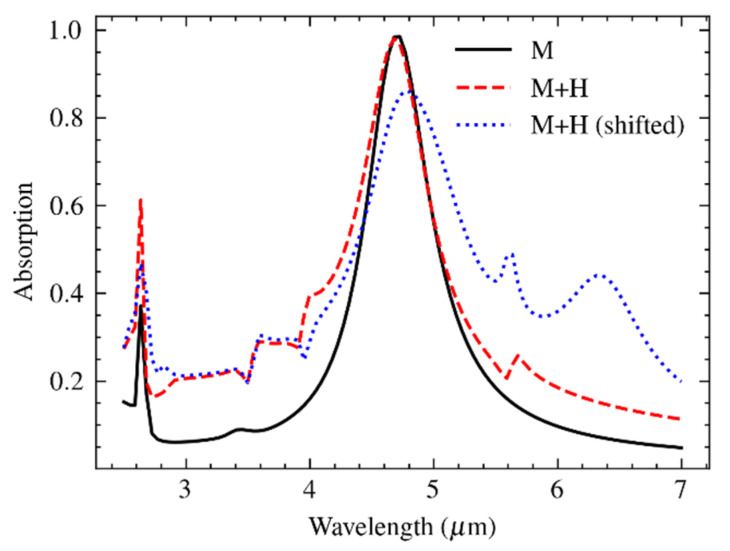
Simulated spectral absorption of the metasurface based on a circular patch of 1.4 μm diameter within a 2.6 μm unit cell and a misaligned array of holes of 2.2 μm diameter and 7.8 μm pitch superimposed in the case of no holes (solid line; M); holes, nominal (dfashed red line; M + H); and over-etched holes and misalignment (dotted blue line; M + H (shifted)).

The results of the different simulations are summarized in Figure 12. The solid line shows the absorption without any hole grid (this is the result shown already in Figure 3). The dashed line shows the effect of the perfectly aligned hole grid, which indicates no significant reduction of the peak absorption, while the out-of-band absorption at the short-wavelength range increases from about 0.08 to 0.2. Adding the deformation due to misalignment results in the dotted line. The most striking features are the higher absorption level in the longer-wavelength out-of-band part of the spectral absorption and the additional peak at about 6.4 μm. This long-wavelength peak was probably due to the strong plasmonic interaction between the hole and unit cell at the points of overlap. The reduced target peak absorbance indicated a decreased in-band effectiveness of the irregularly shaped plasmonic disks. The peak at 2.6 μm wavelength due to PSP resonance broadened when including the array of holes, which may have resulted from the reduced lateral coupling between the plasmonic disks. However, this hypothesis was not validated in this study. 

## 4. Validation and Discussion

**Figure 13:**
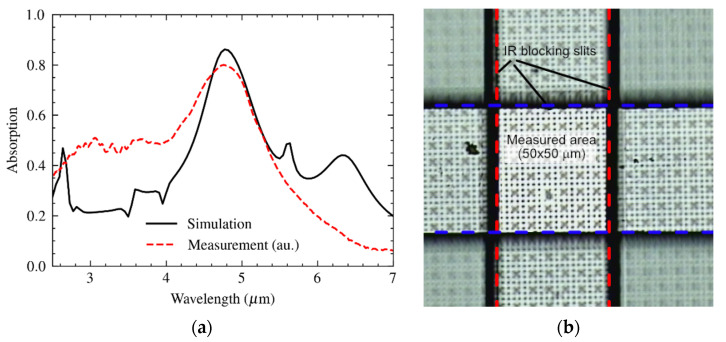
(**a**) Measured spectral absorption of the metasurface based on a circular patch of 1.4 μm diameter within a 2.6 μm unit cell and a superimposed array of holes of 2 μm diameter and 7.8 μm pitch within (**b**) a measurement area as defined by two IR blocking filters, each with a 50 μm wide slit and orthogonally positioned.

The spectra of the fabricated metamaterial absorbers were measured using a liquid N_2_-cooled FTIR spectrometer microscope (Bruker, HYPERION II). A 15× IR-transparent lens was used with a numeric aperture NA = 0.4. This module enabled the measurement of spectral reflectance, *R*(λ), over an area that is defined by two orthogonally placed slits. A gold reflector was used as a reference with an inaccuracy of 1%. When disregarding transmission, the absorbance results from *A*(λ) = 1 − *R*(λ). The result of the spectral measurement is shown in Figure 13a, in comparison to the theoretical prediction (reproduced from Figure 12.

Although the curves show qualitative agreement, there are significant differences. The spectral fine structure was not confirmed. The spectral resolution of the measurement was 1.93 cm^−1^, which is equivalent to 5 nm at about 5 μm wavelength. Consequently, the measurement set-up was not the cause of this spectral smoothening. In earlier work, we observed a significant smoothening of the spectral absorption curve and an enlarged FWHM due to surface roughness of the patches due to the limitations of the metal deposition [11], and the statistical distribution of the dimensions of the unit cells [20] (as in Figure 8a) are likely to also have contributed to this loss in spectral selectivity (there are almost 400 unit cells within the area of the measurement).

Measurements confirmed the out-of-band threshold of the absorption at short wavelengths, which was, however, at a significantly higher level of absorption. This effect was expected to be partially due to the understated transmission through the access holes.

The wavelength of peak absorbance, λ_o_, shifted to longer wavelengths. The slight shift in λ_o_ can be explained when considering the patch diameter, which on average was somewhat larger than the nominal value (see Figure 8b). The variation of patch diameter within the absorber area also resulted in a larger FWHM.

The PSP resonance peak was not present in the measurements, which is possibly due to the lossy medium between the plasmonic disks in addition to the reduced coupling.

The measured absorption at the design wavelength was smaller, as could be expected from simulations, which was due to the assumption of Al_2_O_3_ as a lossless material (*k* = 0). Supplementary measurements, using variable angle spectroscopic ellipsometry (J.A. Woollam M2000) and modeling in WVASE (J.A. Woollam) on a test layer of Al_2_O_3_, indicated that *k* was non-zero but remained significantly lower than *k* = 0.1 up to λ = 1.5 μm (this is the upper limit of the measurement range of the instrument). The literature on the material indicates that *k* remains at very low values up to wavelengths of about 7 μm [36]. Additional simulations indicated a lowering of the peak spectral absorption by about 5% at *k* = 10^−2^ and 30% at *k* = 10^−1^. Therefore, this property increases with *k*, which could explain the reduced peak absorption by about 7% in the measurement. However, we expect that the Al_2_O_3_ layer has additional properties that adversely affect the spectral absorption. The first is the inter-diffusion of Al into Al_2_O_3_, thus resulting in an aluminum-rich lossy dielectric [37]. The second is the roughness of the dielectric surface, which is transferred into the metal patch in the subsequent metal deposition (see process flow in Figure 4). The thickness of the Al top later is about three-times the electron mean free path, and high roughness is known as a cause of a significantly reduced electrical conductivity of such layers [38]. The reduced electrical resistivity with roughness has been reported to significantly affect the spectral absorption [39,40,41]. Supplementary measurements qualitatively confirmed this hypothesis, but further material research is required to fully quantify this effect on the metasurface and to identify approaches for an improved material quality.

## 5. Conclusions

In several ways, the work presented is a study of trade-offs. Earlier work has already shown that several of the known CMOS-compatibility issues are not very limiting in metasurface design and fabrication. The most-widely used dielectric material in silicon microelectronics, SiO_2_, is also a proven material for metasurface operation. However, as shown in this work, a major issue arises when combining surface micromachining with metasurface fabrication. Since SiO_2_ is the sacrificial layer, it was decided to use Al_2_O_3_ for the dielectric layer in the MIM structure instead.

The core question that has prompted this work is how the regular pattern of holes, which is needed for surface micromachining, interferes with the plasmonic response of the metasurface design, and how that interference can be minimized by the dimensions (hole-to-hole pitch and hole diameter), while still ensuring fully under-etched structures. Ideally, this fabrication detail should not be a concern to the optical designer, but that implies that guidelines (design rules) should be formulated. The results of this work indicate that trade-offs are possible in which the access holes can indeed be arranged in such a way that the impact on the spectral absorption of a metasurface for the mid-IR is acceptable. In this work, a hole pitch equal to three times the side length of the unit cell (7.8 μm), and a hole diameter of 2 μm were selected. Although it was clear from the start that this initial choice of especially the access hole diameter is critical from an optical perspective, the adverse effects of a single non-released structure within the large array on overall absorber operation were deemed larger than those of a few damaged unit cells. Therefore, it was decided to use access holes of 2 μm to ensure that fully under-etched structures would be fabricated over the entire large area.

From the results it can be concluded that (a) a pitch of six times the side length of the unit cell is recommendable, and (b) the hole diameter is less critical (Figure 11), but the diameter should be limited to 1–1.5 μm to avoid damaging adjacent unit cells. Obviously, this requirement scales with λ_o_ and becomes more limiting in an absorber designed for operation in the near-IR. It should be noted that although implementation of these measures is realistic, they complicate MEMS process design. A compromise could be sought in the fact that a longer pitch results in fewer damaged unit cells per unit area of metasurface; thus, some margin is available for allowing wider access holes at a longer hole-to-hole pitch. Changing from SiO_2_ to Al_2_O_3_ as the dielectric spacer material, which has become necessary because of surface micromachining, is arguably the most critical constraint of CMOS-compatible MEMS metasurface fabrication.

The surface micromachining process used in this work needs to be optimized in terms of a etch selectivity that would typically allow 1.5 μm diameter access holes at a 13 μm pitch, and also the use of Al_2_O_3_ as the metamaterial dielectric spacer material needs to be reconsidered. Nonetheless, selective coating of the parts of the surface of a silicon wafer that contain devices, such as a thermal detector for use as a bolometer or a resistive heater for use as a hot-wire emitter, as a metasurface is demonstrated to be a viable approach for on-chip integration in a silicon photonics MEMS. Moreover, the feasibility of an optimization strategy between the requirements of the surface micromachining and optical performance of the metasurface is confirmed.

Ongoing work is aimed at a CMOS-compatible process that includes patterning of the sacrificial layer and doping of the poly-Si layers that are used for the thermopile. The intended application is in a micro-spectrometer for selective CO_2_ detection by gas absorption spectroscopy in the 4.0–4.6 μm band in the mid-IR.

## Data Availability

Not applicable.
